# Modeling lymphocyte subset dynamics after ublituximab therapy in patients with multiple sclerosis: an Italian prospective study

**DOI:** 10.3389/fimmu.2025.1688090

**Published:** 2025-11-21

**Authors:** Aurora Zanghì, Paola Sofia Di Filippo, Massimo Papale, Claudia Rutigliano, Maria Claudia Moretti, Carlo Avolio, Gaetano Corso, Emanuele D’Amico

**Affiliations:** 1 Department of Medical and Surgical Sciences, University of Foggia, Foggia, Italy; 2 Clinical Pathology-University-Hospital of Foggia, Clinical Biochemistry, University of Foggia, Foggia, Italy

**Keywords:** Ublituximab, lymphocyte subsets, body mass index, B cells, real-world study

## Abstract

**Background and objectives:**

Ublituximab, a novel, glycoengineered anti-CD20 monoclonal antibody, has recently entered clinical use for multiple sclerosis (MS). In this study, we aimed to delineate the longitudinal kinetics of circulating lymphocyte subsets over the first 6 months following ublituximab initiation. Secondarily, we aimed to investigate whether relevant baseline demographic and clinical characteristics predicted the residual counts at day 30 after infusion of CD3^+^CD8^+^ T cells and CD19^+^ B naive cells, the two subsets that exhibited the most distinctive early kinetics.

**Methods:**

A real-world prospective study was performed at the MS Center of Foggia, Italy. Inclusion criteria were patients with a diagnosis of relapsing MS who started ublituximab between 1 December 2024 and 31 May 2025. Longitudinal trajectories were modeled with subject-specific random-intercept linear mixed-effects models. To identify determinants of early residual depletion, linear regression models were built.

**Results:**

A total cohort of 16 patients was enrolled, with a median age of 47 (Q1–Q3 41–58), 69% men, median EDSS of 4.5, and median body mass index (BMI) of 26.9 kg/m^2^. Mixed-effects models showed a significant effect of time on all lymphocyte subsets. CD3^+^ T cells decreased by 1,577 cells/μL immediately after ublituximab infusion (*p* < 0.001), returning to baseline from day 7 onward. CD3^+^CD8^+^ T cells dropped by approximately 400 cells/μL within the first week (day 7 = 44 cells/μL; 95% CI 11–77) and stabilized from day 30. CD3^+^CD4^+^ T cells fell by 1,133 cells/μL post-infusion (*p* < 0.001), but rebounded from day 7 and remained stable through day 180. CD19^+^ naive B cells remained profoundly suppressed throughout the 6 months (all *p* < 0.001). CD16^+^CD56^+^ NK cells showed a transient reduction of 239 cells/μL at day 0 (*p* = 0.004), normalizing by day 7. Regression analyses at day 30 indicate no significant baseline predictors for CD3^+^CD8^+^ T or CD19^+^ naive B-cell recovery (*R*² = 0.48 and 0.24, all *p* > 0.05). Infusion reactions were mild and self-limited; no adverse events occurred.

**Discussion:**

In our cohort, ublituximab induced rapid, durable CD19^+^ naive B-cell depletion with only transient, reversible effects on other lymphocyte subsets and preserved immunoglobulin levels. This signature extends to older and high BMI patients, supporting ublituximab as a versatile therapeutic option across heterogeneous MS populations.

## Introduction

B cells have recently re-emerged as central players in multiple sclerosis (MS) pathogenesis. Besides contributing to oligoclonal bands synthesis, B cells during MS can also exert antibody-independent processes that include antigen presentation, cytokine production, and formation of ectopic lymphoid follicles within the central nervous system (CNS) (1–3). The advent of anti-CD20 monoclonal antibodies has revolutionized MS management by enabling targeted depletion of B cells, resulting in significant reductions in relapse rates, magnetic resonance imaging (MRI) activity, and disability progression (1, 4–6). Among these agents, ublituximab is a novel, glycoengineered anti-CD20 monoclonal antibody designed to enhance antibody-dependent cellular cytotoxicity (ADCC) and achieve rapid, profound B-cell depletion (7–9).

Personalization of MS therapy increasingly involves the selection of disease-modifying therapies (DMTs) based on individual risk profiles, comorbidities, and biomarkers of disease activity or treatment response. The targeting of CD20^+^ B cells exemplifies this approach, as it allows for the selective modulation of a key pathogenic pathway while sparing other components of the immune system (5). Ublituximab, with its enhanced ADCC and rapid onset of action, offers the potential for swift disease control, which is particularly valuable in patients with highly active or rapidly evolving MS (10).

Despite the efficacy of anti-CD20 therapies, interindividual variability in treatment response remains a clinical challenge. Factors such as body mass index (BMI), age, and baseline immunological status may influence the pharmacokinetics, pharmacodynamics, and immunological effects of these agents (11–14). Higher BMI has been associated with altered drug distribution and potentially reduced efficacy of monoclonal antibodies, while age-related changes in immune function may impact both the safety and effectiveness of B-cell depletion (14). Understanding how these variables affect lymphocyte dynamics and clinical outcomes is essential for optimizing therapy and advancing the goals of precision medicine in MS.

In this study, we aimed to delineate the longitudinal kinetics of the principal circulating lymphocyte subsets over the first 6 months following ublituximab initiation.

We analyzed longitudinal lymphocyte counts with a subject-specific random-intercept mixed-effects model to quantify the 6-month trajectories of CD3^+^ T, CD3^+^CD4^+^ T, CD3^+^CD8^+^ T, CD19^+^ B naive, and CD16^+^CD56^+^ NK cells after the first cycle of ublituximab in a real-world setting. Secondarily, we aimed to investigate whether relevant baseline demographic and clinical characteristics predicted the residual counts at day 30 after infusion of CD3^+^CD8^+^ T cells and CD19^+^ naive B cells, the two subsets that exhibited the most distinctive early kinetics.

## Methods

### Study design and setting

A real-world prospective study was performed at the MS Center of Foggia, Italy. Patients were consecutively admitted between 1 December 2024 and 31 May 2025.

Patients were treated with ublituximab starting from December 2024 through a pre-commercial supply program. The drug was formally classified for hospital-based reimbursement (class H) by AIFA in January 2025 (Italian Official Gazette No/17/22 January 2025).

### Participants and procedures

The inclusion criteria were as follows: 1) patients aged >18 years, with a confirmed diagnosis of RMS, per the revised 2010 McDonald criteria (15); and 2) patients who started ublituximab according to the Italian Medicines Agency prescription rules during the index window.

Ublituximab was administered according to the following regimen: a first intravenous dose of 150 mg, followed by a second infusion of 450 mg after 14 days. Premedication was provided in accordance with the prescribing information to ensure safety and tolerability (16).

The dataset included demographic variables (age, sex, smoking status, BMI, and comorbid conditions) and clinical variables [disease duration, Expanded Disability Status Scale (EDSS) score, naive/switch status to DMTs, number of previously prescribed DMTs, and number of relapses during the year preceding diagnosis]. Baseline brain and spinal cord MRI was performed within 3 months of diagnosis on a 3-T system (Magnetom Skyra, Siemens Healthineers, Erlangen, Germany). The number of gadolinium-enhancing lesions on T1-weighted sequences at baseline MRI was collected.

Brain white matter lesions were segmented by a lesion prediction algorithm (LST toolbox version 3.0.0 for SPM).

Infusion-related adverse events (AEs) were prospectively documented by bedside nurses during each infusion and monitored for the subsequent 24 h as per routine clinical practice. AEs during the follow-up were also collected.

#### Blood sampling schedule

Peripheral blood was obtained at specific time points and was processed within 2 h. Peripheral blood samples were collected at the following 10 time points:

Baseline: prior to treatment start, usually scheduled 3 days before ublituximab first infusion (day −3); immediately after the first split dose infusion of ublituximab (day 0); 1 week after the initial infusion (day 7); before the second split dose infusion of the first cycle (day 14); 1 month after the initial infusion (day 30); 2 months after the initial infusion (day 60); 3 months after the initial infusion (day 90); 4 months after the initial infusion (day 120); 5 months after the initial infusion (day 150); and before the second cycle (day 180).

The normative reference ranges for lymphocyte subsets in our laboratory were as follows: CD3^+^ T cells, 690–2,540 cells/μL; CD3^+^CD4^+^ T cells, 410–1,590 cells/μL; CD3^+^CD8^+^ T cells, 190–1,140 cells/μL; CD19^+^ naive B cells, 90–660 cells/μL; and CD16^+^CD56^+^ NK cells, 90–590 cells/μL.

Serum IgG, IgM, and IgA levels were measured by nephelometry at baseline, day 60, day 30, and day 180.

#### Immunophenotyping

**Figure 1:**
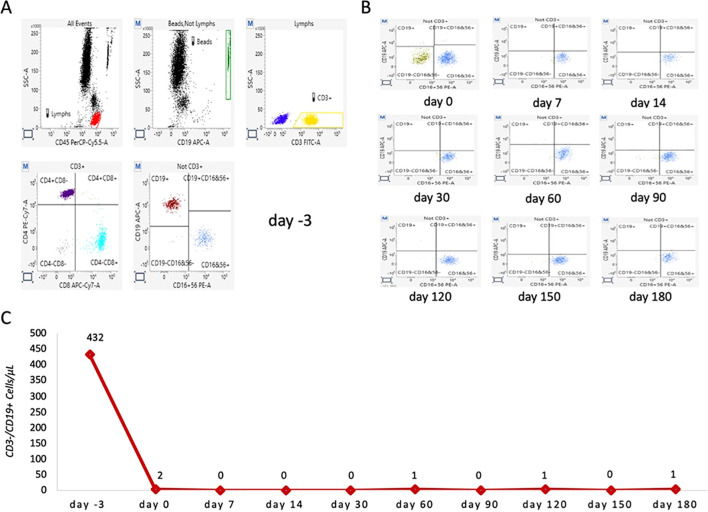
Representative scatter plots of lymphocytes from a studied patient before and after ublituximab infusion. **(A)** Representative baseline lymphocyte profile before ublituximab infusion (day −3). Total lymphocytes (highlighted in red) were gated using side scatter and CD45 PerCP-Cy5.5. CD3^+^ lymphocytes (highlighted in yellow) and CD3^−^ cells (highlighted in blue) were gated by combining side scatter and CD3 FITC. T CD3^+^CD4^+^ (purple) and T CD3^+^CD8^+^ (light blue) lymphocytes were gated from CD3^+^ cells by combining CD4 PE-Cy7 and CD8 APC-Cy7. Finally, CD19^+^ B cells (amaranth) and CD16^+^CD56^+^ NK cells (blue) were gated from CD3-negative lymphocytes by combining CD19 APC and CD16 PE^+^ CD56 PE. **(B)** Representative overview of CD19^+^ B lymphocytes trends immediately after ublituximab infusion (T0) and at 7, 14, 30, 60, 90, 120, 150, and 180 days after infusion. **(C)** Total number of B lymphocytes (CD19^+^) detected at each time point.

Cytofluorimetric analysis was performed using a BD FACSLyric™ flow cytometer (Becton Dickinson - BD, USA) combined with an automated sample preparator (BD DUET system). Cytometer quality control (QC) checks on the optics, fluidics, and electronics were conducted daily using Cytometer Setup and Tracking analysis (CS&amp;T) to ensure reliable and reproducible experimental results (instrumental variability as C.V. was less than 5%). For sample preparation, 10 µL of BD Multitest™ 6-Color TBNK reagent (BD), containing a mixture of six fluorescently labeled monoclonal antibodies (CD3 FITC/CD16 PE + CD56 PE/CD45 PerCP-Cy5.5/CD4 PE-Cy7/CD19 APC/CD8 APC-Cy7), was added to 50 µL of K 3 EDTA whole blood samples in BD Trucount™ tubes and incubated at room temperature for 20 min. Then, 1 mL of lysing solution (Becton Dickinson) was added to each Trucount™ tube and incubated for 10 min at 4 °C–8 °C. Subsequently, the samples were automatically transferred to the instrument for analysis. Data were acquired and analyzed using BD FACSuite™ Clinical software (Becton Dickinson) to obtain the absolute number of each cell type per microliter of blood. T, B, and NK lymphocytes were gated as depicted in **Figure 1**.

### Outcomes and covariate definitions

The primary outcomes of the study were to quantify the 6-month longitudinal kinetics of the major circulating lymphocyte subsets (CD3^+^ T, CD3^+^CD4^+^ T, CD3^+^CD8^+^ T, CD19^+^ naive B, CD16^+^CD56^+^ NK) after the first cycle (two split doses) of ublituximab in patients with relapsing MS (RMS) and to determine the extent and time course of depletion for each subset.

The secondary outcome was to determine whether age, baseline BMI, previous number of DMTs, and the number of gadolinium-enhancing lesions at baseline MRI predicted the residual counts at day 30 of CD3^+^CD8^+^ T cells and CD19^+^ naive B cells, the two subsets that exhibited the most peculiar early kinetics.

### Ethics committee approval and patient consent

Our study adhered to the STROBE (Strengthening the Reporting of Observational Studies in Epidemiology) guidelines for reporting (17). Approval from the local ethical institutional review board was obtained from the Ethical Committee of Bari (7908/2025), and patients signed written informed consent to participate. The study was conducted in accordance with the provisions of the Declaration of Helsinki and the guidelines for good clinical practice set forth by the International Conference on Harmonization.

### Statistical analysis

Categorical variables were reported as proportions. Continuous variables were summarized as mean ± standard deviation (SD) or, when non-normally distributed, as median with interquartile range (IQR). For peripheral–blood lymphocyte subsets, repeated measurements across time points were summarized as mean ± standard error of the mean (SEM).

Absolute counts of the five lymphocyte subsets (CD3^+^ T, CD3^+^CD4^+^ T, CD3^+^CD8^+^ T, CD19^+^ naive B, CD16^+^CD56^+^ NK) were modeled separately with subject-specific linear mixed-effects models.

Day was entered as a categorical fixed effect covering 10 scheduled visits (day −3, day 0, day 7, day 14, day 30, day 60, day 90, day 120, day 150, and day 180 from first infusion); a random intercept captured within-patient correlation, and a variance-component residual structure was retained after Akaike information criterion screening. Fixed-effect significance was evaluated with type-III *F*-tests using Satterthwaite degrees of freedom. Model reliability was summarized by the intraclass correlation coefficient, ICC = σ²_subject/(σ²_subject + σ²_residual). For each visit, estimated marginal means (EMMs) with 95% confidence intervals were extracted; pairwise contrasts were adjusted by the Bonferroni method (two-sided family-wise *α* = 0.05) and reported as adjusted *p*-values.

To identify determinants of early residual depletion, two ordinary linear regressions were constructed at day 30—the time point with maximal data completeness—using log-transformed CD19^+^ naive B cells and CD3^+^CD8^+^ T-cell counts as outcomes. Baseline age, sex, BMI, number of prior DMTs, and number of gadolinium-enhancing lesions at baseline MRI served as predictors. Multicollinearity was excluded (all VIF < 2). Model adequacy was confirmed by normal Q–Q plots, Cook’s distance (<0.5 for all cases), and the Breusch–Pagan test (*p* > 0.10).

All computations were performed in IBM SPSS Statistics, version 27 (IBM Corp., Armonk, NY, USA).

Cell subsets graphics were LOWESS-smoothed (frac = 0.15) in Python 3.11.6 (pandas 2.2.3, statsmodels 0.14.0, matplotlib 3.8.0).

### Data availability

The data that support the findings of this study are available on request from the corresponding author. The data are not publicly available because of information that could compromise the privacy of research participants.

## Results

### Descriptive characteristics of the enrolled cohort

A total cohort of 16 patients completed the first cycle of ublituximab infusion, with 5 (31.2%) female participants, a median age of 47 (41–58), a median EDSS of 4.5 (3.5–5.0), and a median BMI of 26.9 (24.4–28.8). Demographic, clinical, and radiological characteristics of the cohort are shown in **Table 1**.

**Table 1:** Baseline demographic, clinical, and radiological characteristics of the enrolled cohort.

Variable^a^	
Age at ublituximab start (years)	47 (41–58)
Sex, *n* (%)	
*Male*	11 (68.8)
*Female*	5 (31.2)

Comorbidities, *n* (%)	
*Yes*	12 (75)
*No*	4 (25)

Smokers, *n* (%)	
*Yes*	7 (43.8)
*No*	9 (56.2)

Education (years)	13 (8–13)
BMI (kg/m²)	26.9 (24.4–28.8)
Disease duration (months)	21.5 (5.3–153)
Naive to DMTs, *n* (%)	9 (56.3)
No. of prior DMTs	0 (0–2)
No. of relapses within 12 months before enrollment	2 (1–2)
No. of spinal cord lesions at baseline MRI	1 (1–3)
Lesion volume on T2-weighted sequences (baseline MRI) (mm^3^)	2,360.2 (1,484–5,348.7)
No. of patients with gadolinium-enhancing lesions at baseline MRI, *n* (%)	6 (37.5)
EDSS at the time of ublituximab start	4.5 (3.5–5.0)
Time on ublituximab (months)
3.5 (2–5.5)

^a^Data are presented as median (Q1–Q3) for continuous variables.

BMI, body mass index; DMTs, disease modifying therapies; EDSS, Expanded Disability Status Scale; MRI, magnetic resonance imaging; No, number.

Five (31%) patients experienced infusion reactions during the first split dose: transient fever in three cases, self-limited chest tightness with fever in one case, and mild bronchospasm in one case. All events resolved within 24 h with symptomatic treatment and had no sequelae and did not recur during the second split-dose infusion.

No patient developed hypogammaglobulinemia; immunoglobulin concentrations remained within normal reference ranges at every time point (**Supplementary Table 1**). No AEs were recorded during the available follow-up.

### Lymphocyte subset trajectories

The lymphocyte subset data were available for all 16 patients (100%) up to 60 days following therapy initiation. At 90 days, data were available for 11/16 (68.8%) patients. At 120 days, the subset data were available for 8/16 patients (50%). Beyond 120 days, follow-up data were available for 6/16 (37.5%) patients at both 150 and 180 days. These follow-up rates were influenced by the average duration of patient follow-up after starting therapy, reflecting the natural variability in patient retention over time.

**Figure 2:**
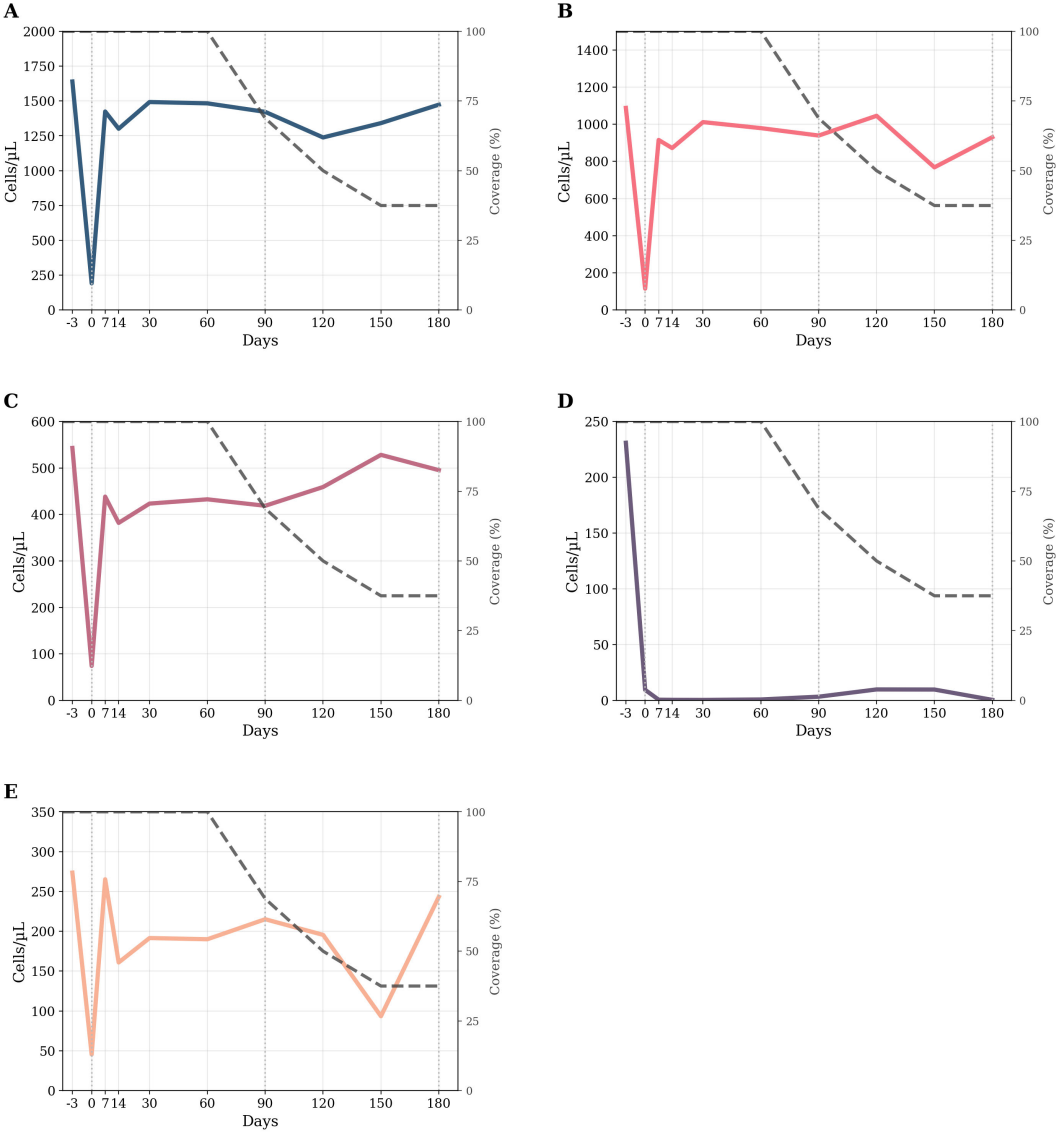
Longitudinal kinetics of circulating lymphocyte subsets over time. **(A–E)** Smoothed (LOWESS, frac=0.15) counts for five lymphocyte subsets from −3 days to 180 days. The colored thick line in each panel tracks the subset’s mean trend; the thin dark-gray dashed line (right axis) reports the actual cohort coverage. Left *y*-axes display absolute cell counts (cells µL−¹); right *y*-axes display coverage percentage. **(A)** CD3^+^ T cells; **(B)** CD3^+^CD4^+^ T cells; **(C)** CD3^+^CD8^+^ T cells; **(D)** CD19^+^ naive B cells; **(E)** CD16^+^CD56^+^ natural killer cells. Legend (common to all panels): –Colored thick line = LOWESS trend for the indicated lymphocyte subset. –Gray thin dashed line = treatment coverage percentage (%). –Vertical dotted lines at 0, 90, and 180 days = reference time points. d = days.

All five lymphocyte subsets displayed a significant effect of time in the mixed-effects models (type-III *F*-tests, all *p* < 0.05). Between-patient stability, as measured by the ICC, varied substantially across cellular subsets, with values ranging from very low to moderate (ICC = 0.017–0.70), indicating that reproducibility was highly dependent on the specific subset analyzed. The amplitude of change ranged from modest fluctuations in total T-cell counts to profound, sustained depletion of CD19^+^ B cells (**Table 2**; **Figure 2**).

**Table 2:** Mixed linear model estimates of peripheral lymphocyte subsets after first ublituximab infusion.

Lymphocyte subset	Model *F* (*df*)1	*p*-valuea	ICC	Baseline Estimated mean (95% CI)	Day 180 Estimated mean (95% CI)	%Δ Baseline→day 180b	*Post hoc*: significant pairwise differences (mean difference* [95% CI], *p*-adj)**
CD3^+^ T cells	6.25 (9, 72.1)	<0.001	0.48	1,639 (1,289–1,990)	1,452 (927–1,976)	−11.6%	Baseline vs. day 0: 1,577 [823, 2,331], *p* < 0.001; all other pairwise comparisons: NS (*p* = 1.000)
CD3^+^CD8^+^ T cells	8.09 (9, 71.2)	<0.001	0.70	505 (427–583)	450.6 (285–616)	−10.8%	Baseline vs. day 0: −406 [−616, −196], *p* < 0.001; baseline vs. day 14: −189 [−369, −9], *p* = 0.03; baseline vs. day 30+: NS (*p* = 1.000)
CD4^+^CD4^+^ T cells	6.89 (9, 71.9)	<0.001	0.56	1,088 (828–1,348)	956 (584–1,328)	−12.1%	Baseline vs. day 0: −1,133 [−1,649.5, −617.2], ≥0.31 all other pairwise comparisons: NS (*p* ≥0.31)
CD19^+^ naive B cells	19.8 (9, 63.9)	<0.001	0.017	231 (200–262)	0.1 (−56 to 56)^c^	−100%	All post-baseline time points (0–180 days) vs. baseline: significant reduction (all *p* < 0.001); no differences among post-baseline (all *p* ≥ 0.99)
CD16^+^CD56^+^ NK cells	2.64 (9, 71.7)	0.011	0.49	274 (183–364)	258 (123−392)	−5.5%	Baseline vs. day 0: −239 [−432.4, −45.0], *p* = 0.004; day 0 vs. day 7: −230.2 [−427.3, −33.1], *p* = 0.008; all others NS (*p* ≥ 0.71)

ICC, intraclass correlation coefficient; NS, not significant.

a Model *F* (*df*): *F* statistic and degrees of freedom from the mixed-effects model.

b %Δ expresses the relative percentage change in cell counts from baseline (day −3) to day 180. Negative values indicate a decrease; positive values indicate an increase.

c Negative values in confidence intervals are statistical artifacts resulting from the estimation process and do not represent actual negative cell counts; these should be interpreted as indicating very low or undetectable levels.

*For *post hoc* pairwise comparisons, the mean difference is reported with its 95% CI and adjusted *p*-value. The sign of the mean difference indicates the direction of change: negative values mean the comparator group has a lower mean than the reference group, and positive values indicate a higher mean. These values reflect the actual differences between groups as estimated by the statistical model.

**Post hoc: Key significant pairwise differences from *post hoc* tests, with mean difference, 95% CI, and adjusted *p*-value.

**Table 2** lists the estimated marginal means at baseline and day 180 and the Bonferroni-adjusted contrasts. The detailed kinetics for each subset are presented in the following section.

#### T cells

##### CD3^+^ T cells

The random-intercept linear mixed-effects model revealed a significant effect of time on absolute CD3^+^ T cells counts (*F* = 6.25, *df* = 9, 72.1, *p* < 0.001). The baseline estimated mean was 1,639 cells µL^−^¹ (95% CI 1,289–1,990), well within the reference interval (690–2,540). Immediately after the first ublituximab infusion (day 0), counts plummeted by 1,577 cells µL^−^¹ (*p* < 0.001), falling far below the lower limit. From day 7 onward, the group mean returned to, and remained within, the physiological range (day 180 = 1,452 cells µL^−^¹; 95% CI 927–1,976). No follow-up value differed significantly from baseline (all adjusted *p* = 1.000). The ICC was 0.48, indicating that 48% of the total variance resided between patients. *Post hoc* pairwise comparisons revealed a highly significant reduction from baseline to day 0 (mean difference: 1,577 [823, 2,331], *p* < 0.001), while all the other time points were not significantly different from baseline (all *p* = 1.000). These findings indicate a profound but rapidly reversible depletion of circulating CD3^+^ T cells after treatment.

##### CD3^+^CD8^+^ T cells

The random-intercept linear mixed-effects model revealed a significant effect of time on absolute CD3^+^CD8^+^ T cells count (*F* = 8.09, *df* = 9, 71.2, *p* < 0.001; ICC = 0.70). Baseline was 505 cells µL^−^¹ (95% CI 427–583), mid-range for the adult reference band (190–1,140). Counts fell by ≈400 cells µL^−^¹ within the first week (day 7 = 44 cells µL^−^¹; 95% CI 11–77) and then re-emerged, but CD3^+^CD8^+^ T cells count remained below baseline with significant differences persisting at day 14 (mean difference: –189 [–369, –9], *p* = 0.03) according to *post hoc* analysis. From day 30 onward, CD3^+^CD8^+^ counts stabilized at the lower end of the normal range and no longer differed significantly from baseline (*p* = 1.000).

The ICC was 0.70, indicating that 70% of the total variance resided between patients. Thus, ublituximab induced a rapid and durable CD3^+^CD8^+^ T-cell depletion that persisted throughout the 6-month observation period.

##### CD3^+^CD4^+^ T cells

The random-intercept linear mixed-effects model also revealed a significant effect of time on absolute CD3^+^CD4^+^ Tcells count (*F* = 6.89, *df* = 9–71.9, *p* < 0.001; ICC = 0.56). Baseline averaged 1,088 cells µL^−^¹ (95% CI 828–1,348), squarely within the normal interval (410–1,590). Immediately post-infusion (day 0), counts dropped by 1,133 cells µL^−^¹ (*p* < 0.001), but rebounded to 915–975 cells µL^−^¹ from day 7 onward and remained stable to day 180. None of the post-infusion values differed from baseline (all adjusted *p* ≥ 0.31), indicating full recovery of the CD3^+^CD4^+^ T compartment after the transient nadir. The ICC was 0.56, indicating that 56% of the total variance resided between patients. These findings indicate a profound but rapidly reversible depletion of circulating CD3^+^CD4^+^ T cells after treatment.

##### CD4^+^/CD8^+^ ratio

Ancillary, the CD4***^+^***/CD8***^+^*** ratio, calculated from raw counts rather than model-derived estimates, climbed from 2 at baseline to 12.8 on day 0, reflecting the near-complete loss of circulating CD3***^+^***CD8^+^ cells and thereafter stabilized (ranged from 2.4 to 2.8) through day 180 (2.2) (data not shown).

##### CD19^+^ naive B cells

The random-intercept mixed-effects model demonstrated a strong time effect on CD19^+^ naive B-cell lymphocyte counts (*F* = 19.8, *df* = 9, 63.9; *p* < 0.001). Median baseline was 231 cells/μL (95% CI 200–262; reference 90–660). Day 0 counts plunged to approximately 9 cells/μL and remained stable at ~0–10 cells/μL throughout the 6 months. Bonferroni-adjusted pairwise comparisons confirmed that every post-baseline time point (0–180 days) differed significantly from baseline (all *p* < 0.001), whereas no differences emerged among post-baseline visits (all *p* ≥ 0.99). The ICC was 0.017, indicating that most variability was within subjects rather than between subjects. Thus, ublituximab induced profound and sustained B-cell depletion.

##### CD16^+^CD56^+^ NK cells

A modest but significant time effect emerged for CD16^+^CD56^+^ NK-cell counts (*F* = 2.64, *df* = 9, 71.7, *p* = 0.011; ICC ≈ 0.49). Baseline averaged 274 cells µL^−^¹ (95% CI 183–364; reference 90–590). Immediately after infusion (day 0), counts dropped to 35 cells µL^−^¹, a reduction of 239 cells µL^−^¹ that remained significant after Bonferroni correction (*p* = 0.004). By day 7, the mean had recovered to 265 cells µL^−^¹, and no subsequent time point differed from baseline (all adjusted *p* ≥ 0.71). The ICC was 0.49, indicating that 49% of the total variance resided between patients. Thus, NK-cell suppression was transient and fully reversible within 1 week.

### Regression analysis of early lymphocyte reconstitution on day 30

The linear regression analysis for CD3^+^CD8^+^ T-cell counts at day 30 after ublituximab infusion showed that none of the baseline variables (sex, age, BMI, number of prior DMTs, gadolinium-enhancing lesions at baseline MRI) significantly predicted early lymphocyte reconstitution (**Table 3**). The model explained 48.1% of the variance in CD3^+^CD8^+^ T-cell counts (*R*² = 0.48), but this was not statistically significant (*F*(5, 5) = 0.93, *p* = 0.532). Among the predictors, age had the largest negative standardized effect (*β* = -0.789), but this was not statistically significant (*p* = 0.122). These findings indicate that early CD3^+^CD8^+^ T-cell recovery at day 30 appears to occur independently of the baseline clinical features examined in this study.

Table 3Linear regression model predicting day 30 CD3^+^CD8^+^ T and CD19^+^ naive B-cell count (cells/µL).

**Table:** 

Day 30 CD3^+^CD8^+^ T cells					
Predictor	Beta (Unstd.)	SE	Beta (Std.)	95% CI (lower, upper)	*p*-value
Intercept	1,434.38	721.98	–	−421.52, 3,290.28	0.104
Sex^a^	182.05	152.43	0.464	−209.79, 573.89	0.286
Age at ublituximab start, years	−13.22	7.12	−0.789	−31.51, 5.07	0.122
BMI (kg/m^2^)	−15.17	24.90	−0.241	−79.17, 48.82	0.569
No. of previous DMTs	−34.85	56.35	−0.220	−179.71, 110.01	0.563
Gadolinium-enhancing lesions at baseline MRI	−98.88	205.60	−0.202	−627.40, 429.65	0.651

Model statistics: *R*² = 0.481; adjusted *R*² = −0.037; *F*(5, 5) = 0.93; *p* = 0.532.

**Table:** 

Day 30 CD19^+^ naive B cells					
Predictor	Beta (Unstd.)	SE	Beta (Std.)	95% CI (lower, upper)	*p*-value
Intercept	−0.51	4.09	–	−11.01, 9.99	0.906
Sex^a^	−0.694	0.863	−0.379	−2.912, 1.523	0.457
Age at ublituximab start years	0.007	0.040	0.083	−0.097, 0.110	0.878
BMI (kg/m^2^)	0.044	0.141	0.148	−0.319, 0.406	0.769
No. of previous DMTs	−0.269	0.319	−0.365	−1.089, 0.550	0.437
Gadolinium-enhancing lesions at baseline MRI	−0.438	1.164	−0.192	−3.430, 2.553	0.722

Model statistics: *R*² = 0.239; adjusted *R*² = −0.522; *F*(5, 5) = 0.31; *p* = 0.885.

BMI, body mass index; DMT, disease-modifying therapies; MRI, magnetic resonance imaging; No., number.

a Male as reference.

The linear regression analysis for CD19^+^ naive B-cell counts at day 30 after ublituximab infusion indicated that none of the baseline variables (age, BMI, number of prior DMTs, gadolinium-enhancing lesions, or sex) significantly predicted early lymphocyte reconstitution (**Table 3**). The model explained 23.9% of the variance in CD19^+^ naive B-cell counts (*R*² = 0.24), but this was not statistically significant (*F*(5, 5) = 0.31, *p* = 0.885). All coefficients were small and not statistically meaningful, with prior DMTs showing the largest negative standardized effect (*β* = -0.365, *p* = 0.437). These findings suggest that early CD19^+^ naive B-cell recovery at day 30 occurs independently of the baseline clinical features examined in this study.

## Discussion

This single-center, prospective, real-world study provides the first description of Italian MS patients treated with ublituximab outside the controlled environment of pivotal phase III trials. Despite its limited size, the cohort mirrors the real-world heterogeneity neurologists encounter, encompassing treatment-naive individuals, patients transitioning from prior DMTs, and an MS population that is progressively aging.

Notably, our study population is characterized by a high median age, a moderate median EDSS, a predominance of male patients, and the frequent presence of spinal lesions—all recognized negative prognostic factors in MS (18, 19). This distinct profile sets our cohort apart from the idealized populations typically enrolled in clinical trials and underscores the strength of our study in evaluating ublituximab in a more challenging, real-world context.

A consistent pharmacodynamic signature emerged almost immediately after the first infusion. Total circulating lymphocytes fell sharply after the first infusion and, with the sole exception of CD19^+^ naive B cells, recovered to baseline by 3 months (CD3^+^CD4^+^ T- and CD3^+^CD8^+^ T-cell counts, CD16^+^CD56^+^ NK), indicating that ublituximab exerts only a transient and reversible effect on non-B-cell lineages (4).

The reversibility of ublituximab’s effects on CD3^+^CD4^+^ T, CD3^+^CD8^+^ T cells, and CD16^+^CD56^+^ NK cells suggests indirect, transient modulation rather than sustained depletion. However, anti-CD20 therapies also target a small subset of CD20^+^ T cells, which display heightened pro-inflammatory and CNS-migratory properties compared to CD20^−^ counterparts (20, 21). Their selective and more durable depletion may partly explain early clinical effects and immune resetting. Recent findings confirm that these cells are preferentially depleted and show distinct repopulation kinetics (22, 23), supporting their relevance as therapeutic targets and their potential role in shaping post-treatment immune profiles.

In contrast, CD19^+^ naive B cells plummeted below 5 cells/µL and remained suppressed throughout the 6-month observation window (5).

Then, ublituximab induces a %Δ from baseline to day 180 of 100% on peripheral CD19^+^ B cells, mirroring the depth and durability reported for other anti-CD20 monoclonal antibodies (e.g., ocrelizumab and ofatumumab) in pivotal and real-world studies (24, 25).

Traditional demographic and radiological modifiers showed minimal influence on either pharmacodynamics or early clinical outcomes. Elevated BMI (mean 26.9 kg/m²) did not blunt B-cell depletion or CD3^+^CD8^+^ T cells, diverging from data derived from other CD20 therapies and also from subgroup phase III Ultimate I and II analyses on ublituximab (11–13, 26).

Likewise, advanced age had no discernible impact on lymphocyte kinetics or infusion-related adverse events. Importantly, no cases of infusion-related anaphylaxis or opportunistic infection occurred, even among patients ≥60 years, echoing the favorable safety profile observed in the pivotal studies.

Compared to other anti-CD20 agents, ublituximab is characterized by a glycoengineered, afucosylated Fc region that markedly enhances affinity for FcγRIIIa and boosts ADCC, independent of receptor polymorphisms. While all anti-CD20 antibodies exhibit similar CD20-binding profiles, ublituximab demonstrates superior effector function *in vitro*, which may explain its rapid and deep B-cell depletion kinetics. These pharmacodynamic features may also influence the broader immune modulation observed in treated patients, beyond B-cell targeting alone (27–29).

Our clinical data suggest that age and BMI did not influence B-cell recovery, although pharmacodynamics dampening associated with immune senescence or obesity is still lacking.

The study has several limitations. Firstly, it is monocentric and involved a modest sample, which constrains external validity. Although linear mixed-effects modeling helped manage missing visits, the small cohort still widens confidence intervals, particularly when exploring predictors of lymphocyte reconstitution. Follow-up ended at 6 months, too short to capture long-term trajectories, and flow cytometry assays were run on a single platform, limiting cross-laboratory comparability. A further limitation of our study is that we performed only generic immunophenotyping of CD3^+^CD4^+^ T and CD3^+^CD8^+^ T cells and CD19^+^ naive B cells, without detailed subtyping of their functional subsets. Unlike previous anti-CD20 studies that have provided a detailed characterization of T, B, and myeloid cell subpopulations—including comprehensive analysis of functional subsets and phenotypic markers—our study lacks this level of immune profiling. As a result, we are unable to directly compare our findings or elucidate the specific mechanisms by which anti-CD20 therapy reshapes the immune network in MS (30–33).

However, these constraints also define areas for future exploration. Larger, multicenter studies integrating extended follow-up and standardized immunophenotyping would allow more granular investigation of interindividual variability and longer-term safety signals, particularly in underrepresented populations (e.g., the elderly or high BMI patients).

Mechanistically, the rapid yet transient decline and recovery of CD3^+^CD4^+^ T, CD3^+^CD8^+^ T, and CD16^+^CD56^+^ NK cells may reflect ublituximab’s selectivity for B cells, with minimal off-target depletion. This reversibility is likely linked to differences in CD20 expression thresholds and repopulation kinetics across immune lineages, with non-B-cell subsets recovering faster than long-lived memory B cells. Further studies are warranted to clarify these dynamics and their relevance for immune homeostasis and clinical outcomes (29).

Notably, the same pattern and favorable short-term safety emerged in challenging subpopulations—older patients, those with higher BMI who are often underrepresented in trials. These findings suggest ublituximab as a valid option across the heterogeneous spectrum of MS care while underscoring the need for broader, longer-term real-world studies to confirm and extend these observations.

## Data Availability

The datasets presented in this study can be found in online repositories. Data will be shared upon reasonable request to corresponding author.
