# Supplementary material for: Modeling lymphocyte subset dynamics after ublituximab therapy in patients with multiple sclerosis: an Italian prospective study
Source: Front Immunol. 2025 Nov 21;16:1688090. doi: 10.3389/fimmu.2025.1688090 (PMC12679708; doi:10.3389/fimmu.2025.1688090)
Supplement: Supplementary file 1 [file DataSheet1.pdf]

Supplementary Table 1. Serum Immunoglobulin Levels at Different Time Points

Immunoglobulin	Baseline (n=16)	Day 60 (n=16)	Day 90 (n=11)	Day 180 (n=6)
**IgG**	915.06 ± 301.35	1005.00 ± 346.35	895.38 ± 224.88	970.40 ± 150.90
**IgA**	163.94 ± 96.38	169.63 ± 107.78	197.13 ± 95.62	212.80 ± 84.52
**IgM**	124.63 ± 53.44	100.88 ± 55.15	106.50 ± 48.98	96.20 ± 44.43

Data are expressed as mean and standard deviation (SD) of serum immunoglobulin G (IgG), A (IgA), and M (IgM) concentrations (mg/dL).

Normative values*: IgG: 700–1600 mg/dL; IgA: 70–400 mg/dL; IgM: 40–230 mg/dL

Kardar G, Oraei M, Shahsavani M, Namdar Z, Kazemisefat G, Haghi Ashtiani M, Shams S, Pourpak Z, Moin M. Reference Intervals for Serum Immunoglobulins IgG, IgA, IgM and Complements C3 and C4 in Iranian Healthy Children. Iran J Public Health. 2012;41(7):59-63. Epub 2012 Jul 31. PMID: 23113211; PMCID: PMC3469020.

(Reference: Adeli K, et al. "Reference intervals for serum immunoglobulins A, G, and M: A practical guide for clinical laboratories." Clin Biochem. 2010;43(7-8): 635-639.)
